# Digital polymerase chain reaction enhances analysis of the cKIT D816V mutation in systemic mastocytosis patients: Clinical insights

**DOI:** 10.1016/j.htct.2025.103938

**Published:** 2025-07-31

**Authors:** Alexandre Eiji Kayano, Luan Lima Marchi, Luciana Nardinelli, Evelin Alves Ramos, Eliane Aparecida Rosseto, Vanderson Rocha, Fernanda Salles Seguro, Elvira Deolinda Rodrigues Pereira Velloso

**Affiliations:** Hematology, Hospital das Clinicas, University of Sao Paulo Medical School, Sao Paulo, Brazil

Systemic mastocytosis (SM) is a rare disease with heterogeneous presentation and the clonal proliferation of mastocytes.^1^, ^2^, ^3^ Over 90 % of cases are well-marked with a molecular signature of the KIT D816V gene mutation.^4^, ^5^, ^6^ However, detecting this mutation depends on the methodology applied, which, to date, lacks standardization, particularly for diagnostic purposes.^6^ Digital polymerase chain reaction (dPCR) is a promising molecular instrument for analyzing low allele burden disease due to its high sensitivity and the absence of a requirement for calibration to achieve quantification. Therefore, many authors advocate its use in SM research and the clinical practice.

The objective of this study was to investigate the correlation between dPCR of the KIT D816V gene mutation and serum tryptase levels, as well as the clinical involvement of disease assessed using the Mastocytosis Activity Score (MAS) and the clinical subtype of SM.

This study involved a prospective analysis of patients diagnosed with SM who were followed-up as outpatients at Hospital das Clínicas, Medicine Faculty of the University of São Paulo between January 2019 and December 2021.

Peripheral blood samples were collected to measure serum tryptase levels using the immunofluorometric method (reference value: up to 14 ug/L), and synchronously genomic DNA was extracted using the QIAamp DNA Blood Midi Kit for the evaluation of the KIT D816V gene mutation via dPCR. The analysis was conducted using the Taqman LiqBiopsy Digital Hs0000000039_rm assay on the QuantStudio3D platform. Each sample was tested twice, with variant allele frequency levels near 0.1 % tested in triplicate. A clinical evaluation and adapted MAS through a one-day interview were conducted by one assistant. SM subtype classification followed 2022 World Health Organization guidelines.^2^

Written informed consent was obtained from all participants in accordance with the Declaration of Helsinki and the protocol was approved by the institutional review board (CAAE number, 29975120.7.0000.0068).

The statistical analysis considered the KIT D816V mutation as the main variable, with serum tryptase levels, MAS and SM subtype as explanatory variables. Fisher’s exact test compared categorical variables by SM subtype, while Pearson’s correlation analyzed gene mutation and continuous variables. Normal distribution was assessed by the Shapiro-Wilk test, and a significance of 5 % was applied to discriminate significant results.

Out of nineteen patients assessed, 31.6 % were classified as having the aggressive subtype, while 68.4 % had the indolent subtype. Additionally, in the aggressive subtype, four out of six were in cytoreductive treatment. Serum tryptase levels were available for 16 patients, with a median of 61.3 µg/L (range: 16.4–200 µg/L). The KIT D816V mutation was detected in 15 out of 19 patients (median: 1.80; range: 0.14–6.79). Additionally, the median of MAS was 12 (range: 0–27).

A significant association was observed between the clinical subtype of SM and serum tryptase levels (p-value = 0.034 - Table 1). Higher values were noted in the aggressive subtype (median: 108.1 µg/L; quintile = 53.8 µg/L) compared to the indolent subtype (median: 51.2 µg/L; quintile = 42.3 µg/L). Furthermore, a modest correlation was found between the KIT D816V mutation and serum tryptase levels (correlation coefficient = 0.52; 95 % confidence interval: 0.03 to 0.08; *p*-value = 0.038 - Table 2).

**Table 1:** . Association between serum tryptase, the KIT D816V mutation and mastocytosis activity score (MAS) stratified by systemic mastocytosis subtype.

	Aggressive subtype	Indolent subtype	*p*-value
	*n* = 6	*n* = 13	
Serum tryptase (µg/L) median (quintile)	108.1 (53.9)	51.3 (42.3)	0.034^a^
KIT D816V mutation median (quintile)^b^	3.52 (2.46)	1.20 (1.00)	0.020^a^
Adapted MAS median (quintile)	11.5 (4.4)	9.2 (8.6)	0.555

a Statistical significance (Fisher exact test <0.05).

b Only those with measurable mutation (*n* = 15) were included.

**Table 2:** Correlation between the KIT D816V mutation with mastocytosis activity score (MAS) and serum tryptase.

	KIT D816V mutation correlation	95 % confidence interval	*p*-value
Adapted MAS	0.45	−0.004 to −0.751	0.052
Serum tryptase	0.52	0.351 to 0.808	0.038^a^

a Statistical significance (Pearson's correlation test <0.05).

A significant association was observed between the KIT D816V mutation and the clinical subtype of SM (*p*-value = 0.02) with higher values noted for the aggressive subtype (median: 3.52; quintile = 2.46) compared to the indolent subtype (median: 1.20; quintile = 1.00). Additionally, a weak correlation was found between the KIT D816V mutation and MAS (correlation coefficient = 0.45; 95 % confidence interval: −0.004 to −0.751; *p*-value = 0.052).

This study revealed a mutation positivity rate of 78.9 % (15 out of 19 patients) using dPCR specific for the D816V locus of the *KIT* gene. The remaining four patients may harbor other mutations (such as V560G, D815K, D816Y, D816F, D816H and D820G) within the same gene, which are found in nearly 5 % of SM cases.

As highlighted before, conventional methods for diagnosing the KIT D816V mutation lack sensitivity. Given its ability to analyze low tumor burden diseases, dPCR has emerged as a promising technique due to this challenge. In a comparison with quantitative PCR, Greiner et al. found a concordance rate of 96 %, indicating non-inferior performance between these two methods for KIT D816V analysis.^7^

The literature currently lacks consensus regarding the optimal sample for detecting the D816V mutation gene. Notably, Greiner et al.^7^ observed a slight advantage for bone marrow samples compared to peripheral blood samples, although this difference was not statistically significant. The present study opted for peripheral blood samples due to their feasibility and the scarcity of literature addressing this issue.

Tumoral burden correlates with serum tryptase levels, which are higher in aggressive subtypes.^1^ This study reaffirms these conclusions and adds the insight of a modest correlation between the KIT D816V mutation and serum tryptase levels, potentially enhancing the assessment of tumor burden.

Hoermann et al.^6^ reported higher values for the KIT D816V mutation in aggressive SM subtypes. However, their study utilized quantitative PCR for this purpose. In this regard, the present study presents pioneering results by employing dPCR to achieve the same goal.

Evaluating and monitoring signs and symptoms in SM is complex. To address this challenge, the MAS has been proposed to standardize medical practice reports and clinical trial assessments.^7^ While a mild correlation was observed between the KIT D816V mutation and MAS in this study, the potential clinical relevance of KIT D816V values exploring symptomatic load underscores the need for a broader cohort study.

It is well known that KIT D816V correlates with multilineage involvement, aggressive subtype, and outcomes.^6^ The current study yielded similar results, with higher variant allele frequency values consistently associated with a more severe clinical burden disease. In conclusion, medical literature currently lacks data on the use of dPCR in SM. We emphasize its value in these patients as it correlates with symptomatic and tumoral burden, measured by adapted MAS/SM subtype and serum tryptase levels, respectively.

## Conflicts of interest

The authors declare no conflicts of interest.
